# Tonic Immobility in PTSD: Exacerbation of Emotional Cardiac Defense Response

**DOI:** 10.3389/fpsyg.2019.01213

**Published:** 2019-05-24

**Authors:** Carlos Eduardo Norte, Eliane Volchan, Jaime Vila, Jose Luis Mata, Javier R. Arbol, Mauro Mendlowicz, William Berger, Mariana Pires Luz, Vanessa Rocha-Rego, Ivan Figueira, Gabriela Guerra Leal de Souza

**Affiliations:** ^1^Institute of Psychology, Universidade do Estado do Rio de Janeiro, Rio de Janeiro, Brazil; ^2^Institute of Biophysics Carlos Chagas Filho, Universidade Federal do Rio de Janeiro, Rio de Janeiro, Brazil; ^3^Department of Clinical Psychology, Universidad de Granada, Granada, Spain; ^4^Department of Psychiatry and Mental Health, Universidade Federal Fluminense, Niterói, Brazil; ^5^Institute of Psychiatry, Universidade Federal do Rio de Janeiro, Rio de Janeiro, Brazil; ^6^Department of Biological Sciences, Universidade Federal de Ouro Preto, Ouro Preto, Brazil

**Keywords:** tonic immobility, cardiac defense response, post-traumatic stress disorder, PTSD, humans, heart rate

## Abstract

Among defensive behaviors, tonic immobility (TI) is considered the last defensive resort when life is at extreme risk. Post-Traumatic Stress Disorder (PTSD) is the main psychiatric consequence resulting from exposure to traumatic events. Increasing evidence indicate an association between peritraumatic tonic immobilility and severity of PTSD. Cardiac defense response, a reactivity to perceived danger or threat, has been studied by recording heart rate changes that follows the presentation of an unpredictable intense auditory aversive stimulus. The aim of this study was to investigate potential distinctiveness in cardiac defense response among PTSD patients who presented – compared to those that did not – TI reaction in the laboratory setting. Patients (*N* = 17) completed the TI questionnaire for signs of immobility elicited by passive listening to their autobiographical trauma script. After a while, they were exposed to an intense white noise, while electrocardiogram was recorded. The heart rate during the 80 s after the noise, subtracted from baseline, was analyzed. Higher reports of TI to the trauma script were associated with stronger and sustained heart rate accelerations after the noise. The effects on cardiac defense response add to increasing evidence that some PTSD patients are prone to repeated re-experiences of TI, which may implicate in a potentially distinct pathophysiology and even a new PTSD subtype.

## Introduction

Post-Traumatic Stress Disorder (PTSD) is the main psychiatric consequence resulting from exposure to traumatic events. It is a frequent, chronic, debilitating, treatment refractory condition (American Psychiatric Association [APA], 2013).

Tonic immobility (TI) is an involuntary reflexive reaction triggered by the perception of inescapable danger, characterized by reversible profound motor inhibition and relative unresponsiveness to external stimuli (Ratner, 1967). Systematic studies with TI scales based on retrospective reports, which were further corroborated with objective biological recordings (see Volchan et al., 2017); pointed to the presence of TI in humans. Although the precise underlining subtracts of human TI has yet to be identified, biological indicators using posturography and electrocardiography recordings were successfully reported, namely reduced body sway, tachycardia and low heart rate variability (Volchan et al., 2011). Importantly, experimental induction of TI using script-driven symptoms provocation was more evident in patients with PTSD than in trauma-exposed controls (Volchan et al., 2011, 2017). For retrospective reports, in a large epidemiological sample, greater TI scores were found to be associated with PTSD (Kalaf et al., 2015). Other studies observed that retrospective reports of peritraumatic TI are correlated with the severity of PTSD symptoms (Fiszman et al., 2008; Rocha-Rego et al., 2009; Lima et al., 2010; Portugal et al., 2012; Maia et al., 2015; Kleine et al., 2018).

Cardiac defense response can be defined as the cardiac reactivity to perceived danger or threat. Recording heart rate changes and the presentation of an unpredictable intense auditory aversive stimulus, studies showed two accelerative-decelerative components: a brief acceleration reaching maximum amplitude around second 2-3 followed by a fast deceleration and then a more sustained acceleration followed by a final deceleration, the whole pattern lasting up to 80 s (Vila et al., 2003; Vila et al., 2007). Under a pre-existing aversive emotional state – people with generalized anxiety disorder, chronic worry, or with specific phobia having visualized the phobic object prior to the intense auditory stimulus – the response pattern changes to a single sustained accelerative response without the initial deceleration (Vila et al., 2007; Delgado et al., 2009). Schalinski et al. (2013) investigated the cardiac defense response in women war refugees. Those with PTSD showed sustained accelerative response pattern without the initial deceleration, while those without PTSD showed the initial acceleration-deceleration followed by a reduced second acceleration-deceleration.

Our aim was to investigate potential distinctiveness in cardiac defense response among PTSD patients who presented – compared to those that did not – TI reaction after re-experiencing the trauma in the laboratory setting. Based on our previous observations of robust tachycardia under scrip-driven paradigm (Volchan et al., 2011), we hypothesized that subsequent exposure to an intense aversive sound would evoke, in PTSD patients reporting TI during script-driven symptoms provocation, a response pattern characterized by sustained acceleration significantly different from the response evoked by PTSD patients not reporting TI. The latter would show the initial acceleration-deceleration followed by a reduced second acceleration-deceleration.

## Materials and Methods

### Participants

Victims of urban violence diagnosed with PTSD (7 men, 10 women) participated in the study. They were under treatment with selective-serotonin-reuptake-inhibitors and in the waiting list for cognitive-behavior-therapy in the Outpatient University Clinic specialized in PTSD. Psychiatric diagnoses were obtained using the DSM- IV Axis I (First et al., 1997; Del-Ben et al., 2001). Exclusion criteria were psychotic disorders, severe personality disorders, use of Haloperidol, and significant cognitive impairment. For more information about the sample, see Table 1.

**Table 1:** Socio-demographic, trauma-related and clinical characteristics of the sample (*n* = 17).

**Age**	
Mean (SD) (in years)	41.1 (6.43)
**Marital status**	
Married	62.5%
Single	25%
Divorced	6.3%
Living maritally	6.3%
**Occupation**	
White collar	37.5%
Blue collar	62.5%
**Educational level**	
College graduate	18.8%
Some college	12.5%
High school graduate	18.8%
High school dropout	18.8%
Elementary school graduate	18.8%
Elementary school dropout	12.5%
**Type of traumatic event**	
Armed robbery	31.3%
Traffic accidents	18.8%
Shooting	18.8%
Rape	12.5%
Being kidnapped	12.5%
Other	6.3%
**Childhood abuse**	
Sexual	6.3%
Physical	6.3%
**PCL-C**	
Mean score (SD)	66.8 (9.94)
**BDI**	
Mean score (SD)	30.3 (12.1)
**Current psychiatric comorbidities**	
Mean number (SD)	4.0 (1.59)
Major depressive disorder	87.5%
Obsessive-compulsive disorder	75%
Panic disorder and/or agoraphobia	37.5%
Social anxiety disorder	37.5%
Specific phobia	37.5%
Substance use disorder	18.8%

*PCL-C, post-traumatic stress disorder checklist. BDI, beck depression inventory.*

The study was approved by the local Ethics Institutional Review Board and participants provided written informed consent.

### Psychometric Assessment

Based on their index trauma, participants completed the PTSD Checklist-Civilian Version (PCL-C for DSM IV) (Weathers et al., 1993; Berger et al., 2004).

Based on TI Scale (TIS-C: Forsyth et al., 2000), we used a four-item measure of motor aspects of TI which has been validated (Lima et al., 2010) and employed in previous studies (Rocha-Rego et al., 2009; Volchan et al., 2011; Portugal et al., 2012): (i) froze or paralyzed; (ii) unable to move; (iii) unable to call out or scream, and (iv) unable to escape. Total scores range from zero to 24. Cronbach alpha for this sample was 0.88.

### Stimulus

The auditory stimulus was an intense white noise of 105 dB with 500 ms duration and instantaneous rise time, delivered binaurally throughout earphones (Eartone). Given the fast habituation of the cardiac defense response (Ramírez et al., 2010), the stimulus was presented just once.

### Data Acquisition

Two PC-compatible computers controlled auditory stimulus presentation and data acquisition of the electrocardiographic parameters, running Presentation (Neurobehavioral Systems) and Acknowledge (BIOPAC Systems Inc.) softwares. Electrocardiographic recordings were collected at a sampling frequency of 1000 Hz through an electrocardiograph ECG100C module coupled to the MP150 system (BIOPAC Systems Inc.).

### Procedures

A psychiatrist accompanied the patient throughout the experimental session and debriefing. Participants had electrocardiogram electrodes attached on the chest at lead II and wore earphones. Posturography was recorded while participant stood on a force platform and, after a 60s-neutral playback, they listened to a 60s-audio-play narrating his/her personal traumatic script, Then, participant was seated and the psychiatrist applied the TI questionnaire to assess signs of immobility elicited by the trauma script (for more details and cardiac and postural results, see Volchan et al., 2011). After relaxing for 25 min, they were exposed to the auditory stimulus, while electrocardiogram was recorded.

### Data Reduction

An off-line peak detection algorithm (derivative plus threshold) was used to estimate R-wave fiducial points, after which the series was screened by hand and corrected for artifacts. R–R intervals were converted to heart rate each second using weighted averages during the 80 s following stimulus onset and expressed as differential score subtracting the weighted average of the 15 s prior to stimulus onset. Data processing followed the recommendations of the Task Force of the European Society of Cardiology and the North American Society of Pacing and Electrophysiology (1996) concerning the measuring of heart rate instead of heart period when using time units (second-by-second) versus organismic units (beat-by-beat), accompanied by weighted averages versus arithmetic averages within the time window (each second). Previous studies used to further reduce the 80 second-by-second heart rate values to 10 intervals (Vila et al., 2007) or 12 intervals (Schalinski et al., 2013). Given that current statistical software allows the analysis of long time series, as repeated measures, the present study maintained the 80 heart rate values, without further reduction, in order to fully describe the response pattern.

### Statistical Analyses

Sum scores in the four motor questions of the TI scale were calculated for each participant. Those with scores higher than the sample’s median were assigned to the “HIGH” group and those with scores equal or lower than the median, to the “LOW” group. We used Student’s *t*-test for the comparisons of TI scale, PCL scale and Baseline Heart Rate immediately before the delivery of the sounds between groups. A 2 x 80 ANOVA with one between group factor (Group) and one repeated measures factor (Time) was employed to compare the heart rate data between groups. The Greenhouse-Geisser correction was applied to control for sphericity violation in the repeated measures factor.

Pearson’s correlation analyses were also performed between heart rate and TI scores for the whole sample.

The results are reported with the original degrees of freedom, the corrected *p* values, and the effect sizes. The threshold for significance was set at *p* ≤ 0.05.

## Results

### Psychometric Assessment

Based on scores in the four motor questions of TI elicited by listening to the personal trauma script, 8 participants (4 women) were ascribed to the “HIGH” and 9 (6 women) to the “LOW” group. Scores differed significantly between the groups (HIGH: 17.9 ± 5.57; LOW: 3.1 ± 3.18; *t*_(15)_ = 6.82, *p* < 0.01). Comparison of PTSD symptoms, based in scores on the PCL-C scale, between “HIGH” and “LOW” groups were not significant (HIGH: 61.1 ± 9.23; LOW: 54.7 ± 13.66; *t*_(15)_ = 1.13, *p* = 0.27).

### Heart Rate

**FIGURE 1:**
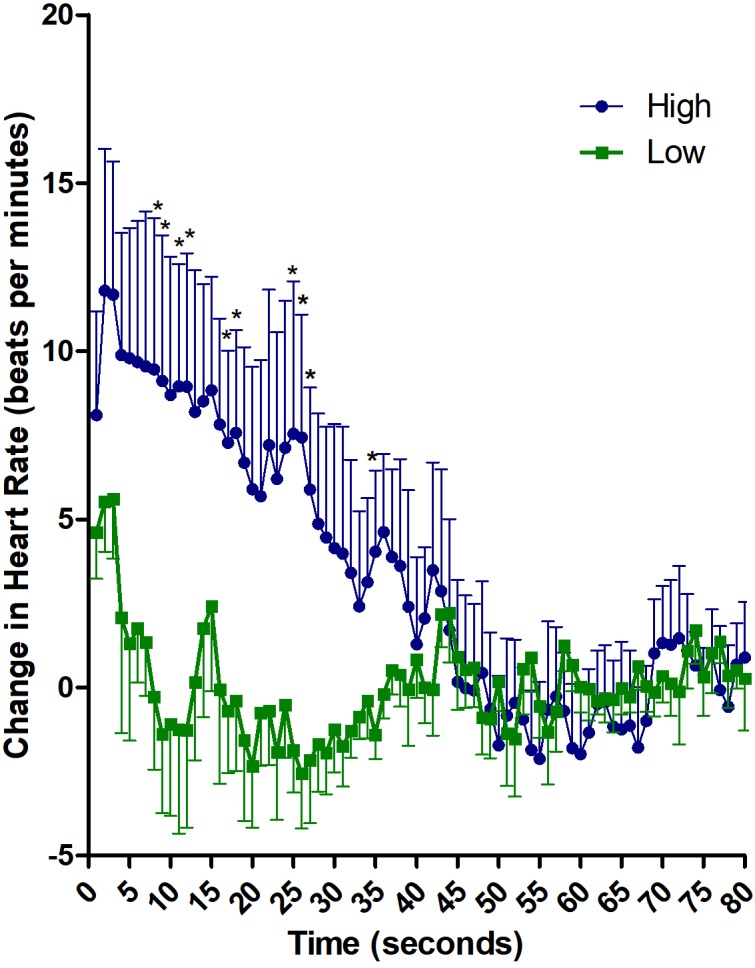
Descriptive analysis of cardiac defense response in HIGH and LOW tonic immobility patients. Heart rate changes along 80 s after the noise stimulus, subtracted from mean baseline. ^∗^*p* ≤ 0.05 in seconds 8, 9, 11, 12, 17, 18, 25, 26, 27, and 35.

Baseline heart rate before the delivery of the sound did not differ between “HIGH” and “LOW” groups (HIGH: 80.3 ± 16.7; LOW: 71.2 ± 15.3; *t*_(15)_ = 1.18, *p* = 0.26). Descriptive analysis of heart rate (subtracted from baseline), after the exposure to the aversive noise, showed a larger and longer accelerative response in the HIGH than in the LOW TI group (Figure 1). The peak of the acceleration occurs at second 3 after stimulus presentation. The LOW TI group presented a fast decrease afterward reaching decelerative values after second 7, whereas the HIGH TI group showed a sustained acceleration that lasted until second 45.

The 2 x 80 ANOVA revealed significant effects of Time [*F*_(79,1185)_ = 4.66, *p* < 0.01; ηp^2^ = 0.237] and Group x Time [*F*_(79,1185)_ = 3.78, *p* < 0.01; ηp^2^ = 0.201]. Follow-up analysis of the interaction revealed significant differences between HIGH and LOW immobility groups in the first 30 s after stimulus onset [*F*_(1,15)_ = 4.44, *p* = 0.05; ηp^2^ = 0.228]. Average increase in heart rate in the first 30 s was 7.88 ± 10.1 for HIGH group and 0.03 ± 4.5 for LOW group. When the heart rate was analyzed second-by-second, significant differences between the groups appeared in seconds 8, 9, 11, 12, 17, 18, 25, 26, 27, and 35 (*p* ≤ 0.05; Cohen’ *d* ≥ 1.0 for all comparisons). Further, correlation analyses between heart rate and TI scores was significant for average heart rate in the first 30 s (*r* = 0.491; *p* = 0.04) and for seconds 6, 8, 9, 10, 11, 12, 17, 20, 25, 26 and 35 (*r* ≥ 0.49; *p* ≤ 0.05 for all comparisons).

## Discussion

This is the first study to investigate the modulation of the cardiac defense response in PTSD patients after the induction of a TI-like reaction in a laboratory setting. PTSD patients who reported high levels of immobility after listening to their autobiographical trauma exhibited, upon exposure to the unexpected and intense auditory stimulus, a more pronounced and sustained accelerative cardiac defense response than patients who reported low levels or did not report at all signs of immobility. Comparisons of PTSD symptoms scores between those with high and low scores of TI did not reach statistical significance, which favors TI as a main factor differentiating sustained versus transient tachycardia in response to the aversive sound.

Recent research has been looking for the relationship between defensive reflex responses and psychopathology, investigating if different patterns of responsiveness can be associated with clinical conditions (McTeague and Lang, 2012). Contributing with the RDoC initiative, Lang et al. (2016) suggests a complex and dynamic modulation of defensive reactivity across the anxiety disorder spectrum by physiological responses.

Schauer and Elbert (2010) proposed stages of defensive reactions in which TI, accompanied by the highest sympathetic activation, is at the peak of the fright stage. Alves et al. (2014) showed in victims of urban violence that high scores on peritraumatic TI were associated with tachycardia during exposure to trauma-relevant pictures. Volchan et al. (2011) showed that reports of TI after trauma-script listening were inversely correlated with amplitude of body sway and with heart rate variability, and directly correlated with heart rate; specially for the urban-violence victims with PTSD (Volchan et al., 2017). The authors concluded that peritraumatic TI was experienced in the laboratory setting and was accompanied by tachycardia and lowering of vagal tone.

In the present study, PTSD patients displaying peritraumatic TI after exposure to trauma-script presented a pattern of cardiac defense response with more accelerative and sustained cardiac response indicating that they are more prone to cardiac sympathetic activation in response to varied aversive intense stimuli accompanied by reduced influence of the parasympathetic system.

This distinctiveness adds to a growing literature concerning peritraumatic TI symptoms which might indicate a specific PTSD subtype responding differently to pharmacological treatment (Fiszman et al., 2008; Lima et al., 2010) and deserving the development of alternative interventions based on a potentially distinct pathophysiology. Still, the present sample was small and PTSD patients were under medication. Large scale randomized controlled trials are necessary to corroborate the promising findings.

## Ethics Statement

The study was approved by the Ethics Institutional Review Board of the Institute of Psychiatry (Federal University of Rio de Janeiro). Written informed consent was provided by all participants, in accordance with the Declaration of Helsinki.

## Author Contributions

CN, EV, VR-R, IF, GdS, MM, WB, and JM developed the study concept and the study design. CN, GdS, WB, and ML conducted the experimental sessions. CN, JM, JA, JV, GdS, and EV performed data analysis. GdS, CN, VR-R, and EV drafted the manuscript. IF, WB, MM, ML, and JV provided important critical revisions. All the authors approved the final version of the manuscript and agreed to be accountable for all aspects of the work.

## Conflict of Interest Statement

The authors declare that the research was conducted in the absence of any commercial or financial relationships that could be construed as a potential conflict of interest. The reviewer MP declared a past co-authorship with one of the authors (JV) to the handling Editor.
